# Repeat-mediated epigenetic dysregulation of the *FMR1* gene in the fragile X-related disorders

**DOI:** 10.3389/fgene.2015.00192

**Published:** 2015-06-03

**Authors:** Karen Usdin, Daman Kumari

**Affiliations:** Section on Gene Structure and Disease, Laboratory of Cell and Molecular Biology, National Institute of Diabetes, Digestive and Kidney Diseases, National Institutes of Health, Bethesda, MD, USA

**Keywords:** fragile X-associated tremor/ataxia syndrome, fragile X-associated primary ovarian insufficiency, fragile X syndrome, Repeat-mediated gene silencing, R loops, polycomb repressive complex 2

## Abstract

The fragile X-related disorders are members of the Repeat Expansion Diseases, a group of genetic conditions resulting from an expansion in the size of a tandem repeat tract at a specific genetic locus. The repeat responsible for disease pathology in the fragile X-related disorders is CGG/CCG and the repeat tract is located in the 5′ UTR of the *FMR1* gene, whose protein product FMRP, is important for the proper translation of dendritic mRNAs in response to synaptic activation. There are two different pathological *FMR1* allele classes that are distinguished only by the number of repeats. Premutation alleles have 55–200 repeats and confer risk of fragile X-associated tremor/ataxia syndrome and fragile X-associated primary ovarian insufficiency. Full mutation alleles on the other hand have >200 repeats and result in fragile X syndrome, a disorder that affects learning and behavior. Different symptoms are seen in carriers of premutation and full mutation alleles because the repeat number has paradoxical effects on gene expression: Epigenetic changes increase transcription from premutation alleles and decrease transcription from full mutation alleles. This review will cover what is currently known about the mechanisms responsible for these changes in *FMR1* expression and how they may relate to other Repeat Expansion Diseases that also show repeat-mediated changes in gene expression.

## Introduction

The fragile X-related disorders are members of 20+ human genetic conditions known as the Repeat Expansion Diseases (Mirkin, 2007). The disease-causing mutation in all cases is an expansion or increase in the number of repeats in a specific tandem repeat tract. In the case of the fragile X-related disorders, the repeat unit is CGG/CCG and the repeat tract is located in the 5′ untranslated region of the fragile X mental retardation 1 (*FMR1*) gene (Fu et al., 1991; Verkerk et al., 1991). The *FMR1* gene encodes FMRP, a protein important for the regulation of translation of dendritic mRNAs in response to synaptic activation (Weiler et al., 1997; Weiler and Greenough, 1999). Two different pathological *FMR1* allele size classes are distinguished. Premutation alleles have 55–200 repeats, while alleles with >200 repeats are referred to as full mutation alleles. These two size classes are distinguished because they confer risk of different clinical conditions. Carriers of premutation alleles are at risk of an adult-onset neurodegenerative disorder known as fragile X-associated tremor/ataxia syndrome (reviewed, in Hagerman, 2013). Female carriers are also at risk of fragile X-associated primary ovarian insufficiency, a condition that is associated with fertility problems and an earlier than normal menopause (reviewed in, Sullivan et al., 2011; Sherman et al., 2014). In contrast, full mutation alleles are associated with fragile X syndrome, the leading heritable cause of intellectual disability and the major monogenic cause of autism (Oberle et al., 1991; Fu et al., 1991; Verkerk et al., 1991).

Premutation and full mutation carrier symptoms are different, in part, because the repeat has paradoxical effects on gene expression. Premutation alleles are hyper-expressed (Tassone et al., 2000a) and disease symptoms result from the deleterious consequences of high levels of expression of the transcript with large numbers of CGG-repeats (reviewed, in Sellier et al., 2014). In contrast, the symptoms of fragile X syndrome result from repeat-mediated heterochromatin formation that causes transcriptional silencing and a subsequent deficiency of FMRP (Pieretti et al., 1991; Sutcliffe et al., 1992). Interestingly, it is becoming increasingly apparent that heterochromatin formation is a common feature of other Repeat Expansion Diseases that involve large repeat tracts. This includes myotonic dystrophy type 1, Friedreich ataxia and amyotrophic lateral sclerosis/frontotemporal dementia (ALS/FTD; Otten and Tapscott, 1995; Steinbach et al., 1998; Herman et al., 2006; Greene et al., 2007; Belzil et al., 2013; Xi et al., 2013).

How the repeats cause hyper-expression of premutation alleles and silencing of full mutation alleles is not well understood. This review will cover recent findings that suggest how the repeats are able to have such different effects on gene expression.

## The CGG-repeat Tract Forms Stable Secondary Structures

The CGG-repeat that is present in both the *FMR1* gene and its transcript can form a variety of secondary structures. *In vitro* the DNA repeats form a stem-loop/hairpin and a folded-hairpin-like structure known as a G-tetraplex or quadruplex (Fry and Loeb, 1994; Kettani et al., 1995; Mitas et al., 1995; Usdin and Woodford, 1995; Patel et al., 2000). The hairpin contains a mixture of Watson-Crick G:C base pairs and Hoogsteen G:G base pairs in a 2:1 ratio. The quadruplex is stabilized primarily by G-quartets. The RNA forms a similar set of structures (Handa et al., 2003; Zumwalt et al., 2007; Malgowska et al., 2014). Similar structures are also formed by many of the repeats responsible for other members of the Repeat Expansion Disease family that show repeat-mediated epigenetic changes (reviewed, in Usdin et al., 2015), and quadruplex formation has been reported for the very GC-rich ALS/FTD repeats (Fratta et al., 2012; Reddy et al., 2013; Haeusler et al., 2014) and the repeats responsible for progressive myoclonus epilepsy type 1 (EPM1; Saha and Usdin, 2001). There is evidence for the formation of stem-loop structures *in vivo* by the CGG-strand of the fragile X repeat (Loomis et al., 2014) and the CAG- and CTG-repeats responsible for diseases like Myotonic dystrophy type 1 and Huntington disease (Liu et al., 2010). Transient unpairing of the repeat region during DNA replication, DNA repair or transcription is thought to provide the opportunity for these structures to form.

In addition to the above-mentioned structures, many disease-associated repeats including the fragile X repeats (Groh et al., 2014; Loomis et al., 2014), the Friedreich ataxia repeat (Groh et al., 2014), the ALS/FTD repeat (Haeusler et al., 2014) and the CTG/CAG-repeats responsible for a number of Repeat Expansion Diseases (Lin et al., 2010) form stable R loops during transcription. This R loop contains an RNA:DNA hybrid formed between the nascent transcript and the transcribed strand. This leaves the displaced non-template strand unpaired. Such R loops form in regions with strand-asymmetry with regard to the distribution of purines and pyrimidines, particularly when the pyrimidine-rich strand is being transcribed. This R loop may be facilitated by hairpin formation by the non-template strand that would reduce the likelihood of reannealing of the duplex behind the advancing transcription complex. Conversely, the persistence of this hybrid might also favor the formation of the hairpin on the non-template strand. R loops are the only structures identified to date that are formed by all repeats that become heterochromatinized. In bacteria and yeast R loops can also be formed *in trans* (e.g., by a distally transcribed mRNA) in the presence of RecA and Rad51 respectively (Zaitsev and Kowalczykowski, 2000; Wahba and Koshland, 2013), but evidence for such R loops in mammalian cells is lacking.

The number of repeats required to form an R loop on the *FMR1* gene and the timing of R loop formation is the subject of some debate. One study suggests that they are only formed on full mutation alleles relatively late in neuronal differentiation (Colak et al., 2014), while other work suggests that R loops at the *FMR1* locus also form on normal and premutation alleles in a number of different cell types (Groh et al., 2014; Loomis et al., 2014). This discrepancy may reflect differences in the stability of the structures being measured. Formation of these structures *in vivo* likely reflects some combination of the effect of repeat number, transcription rate (Groh et al., 2014; Loomis et al., 2014) and the expression of proteins that affect R loop stability (Colak et al., 2014).

## Repeat-mediated Epigenetic Effects on the Premutation Allele

There is a direct relationship between the repeat number and *FMR1* mRNA levels in humans and mice carrying the premutation allele, such that premutation carriers have 2–10 times more *FMR1* mRNA than individuals with repeat numbers in the normal range (Tassone et al., 2000b; Entezam et al., 2007; Brouwer et al., 2008). The increased RNA levels are the result of increased transcription initiation rather than increased transcript stability (Tassone et al., 2007). The promoter of premutation alleles is enriched for acetylated histones (Todd et al., 2010) and premutation alleles initiate transcription from upstream start sites more frequently than is seen in normal cells (Beilina et al., 2004). While hyper-expression has not been reported for other Repeat Expansion Diseases, a similar increased usage of more 5′ start sites has been reported in individuals with ALS (Sareen et al., 2013).

Very little is currently known about why premutation alleles are overexpressed. CGG/CCG-repeats exclude nucleosomes *in vitro* (Wang et al., 1996). In principle, this could result in the 5′ end of the *FMR1* gene being more accessible to transcription factors or to the transcription complex. However, to date there is no evidence for repeat-mediated nucleosome exclusion on *FMR1* alleles *in vivo*. The *FMR1* promoter is very CpG rich and has many of the hallmarks of a CpG-island promoter. CpG-island promoters act as transcription-independent nucleation sites for the zinc finger CxxC domain-containing chromatin modifying proteins like CxxC finger protein (CFP1; Thomson et al., 2010). CFP1 is a component of the SET1A/B-containing methyltransferase complex that facilitates H3K4 trimethylation (Clouaire et al., 2012), a histone mark typically associated with the 5′ ends of active genes. This has led to the suggestion that such proteins provide a self-reinforcing loop of unmethylated CpG recognition and subsequent protection from DNA methylation (Blackledge et al., 2013). In this view, hyper-expression of premutation alleles would be related to the high density of CpGs in the repeat that favors recruitment of factors that inhibit gene silencing. Alternatively, proteins like ATRX, a member of the SNF2 family of helicases/ATPases, could also contribute to hyper-expression of premutation alleles. ATRX colocalizes with G-rich regions that, like the *FMR1* locus (Fry and Loeb, 1994; Kettani et al., 1995; Usdin and Woodford, 1995; Patel et al., 2000), have quadruplex-forming potential. ATRX facilitates transcription elongation through these regions by reducing transcription stalling (Levy et al., 2015).

R loops are a characteristic feature of unmethylated CpG-islands where they have been suggested to play a role in preventing gene silencing (Ginno et al., 2012). There is evidence to suggest that R loops provide some protection from *de novo* methylation by DNMT3B1, the primary *de novo* DNA methyltransferase active during early development (Ginno et al., 2012). How they do so is currently unknown, but one possibility is that the single stranded region of the R loop is a preferential binding site for a number of epigenetic modifiers that are positive regulators of transcription. These modifiers include members of the H3K4 methyltransferase family (Krajewski et al., 2005), whose activity is thought to inhibit *de novo* methylation (Ooi et al., 2007). It also includes the activation-induced cytosine deaminase (Chaudhuri et al., 2003) that is thought to be important for DNA demethylation (Popp et al., 2010). R loops may also be less capable of properly binding nucleosomes (Dunn and Griffith, 1980) and there is evidence to suggest that R loop formation causes chromosome decondensation (Powell et al., 2013). R loops formed on longer repeat tracts have been shown to extend further into the flanking regions (Loomis et al., 2014). This could perhaps favor transcription initiation by increasing the likelihood that promoter melting will occur or by facilitating the binding of additional transcription factors or chromatin modifiers to the promoter that in turn promote transcription initiation.

## Repeat-mediated Gene Silencing of Full Mutation Alleles

In contrast to the marks of active chromatin found at the 5′ end of the *FMR1* gene in premutation carriers, full mutation alleles are hypermethylated and associated with hypoacetylated histones (Chiurazzi et al., 1998, 1999; Coffee et al., 1999; Pietrobono et al., 2002, 2005; Biacsi et al., 2008). They are also hypomethylated on H3K4, enriched for marks typical of facultative heterochromatin like H3K9me2 and H3K27me3, as well as histone modifications associated with constitutive heterochromatin- H3K9me3 and H4K20me3 (Kumari and Usdin, 2010). H3K9 dimethylation has also been reported on Myotonic dystrophy type 1 alleles (Cho et al., 2005), H3K9 trimethylation has been reported on Friedreich ataxia alleles (Herman et al., 2006) and H3K9, H3K27, and H4K20 trimethylation have been reported for heterochromatinized ALS alleles (Belzil et al., 2013).

Treatment of fragile X patient cells with DNA methylation inhibitors leads to partial gene reactivation that is not associated with the loss of H3K9me2 or H3K9me3 (Kumari and Usdin, 2014). This suggests that DNA methylation occurs downstream, or is independent of, the deposition of these chromatin modifications. This would be consistent with the observation that in rare full mutation carriers in which gene silencing does not occur, the *FMR1* gene is enriched for H3K9me2 but shows no CpG methylation (Tabolacci et al., 2008). The *FMR1* gene can also be reactivated by inhibition of Sirtuin 1 (SIRT1), the enzyme responsible for the deacetylation of H3K9 and H4K16 on fragile X alleles (Biacsi et al., 2008). Since DNA demethylation leads to acetylation of H4K16 but not H3K9 (Biacsi et al., 2008), it would be consistent with the idea that deacetylation of H3K9 precedes DNA methylation while deacetylation of H4K16 is a late event in the silencing process acting downstream of DNA methylation.

The earliest epigenetic change associated with silencing of the fragile X allele is unknown. However, the fact that the histone marks H3K9me3 and H4K20me3 show a peak of enrichment in the region of the fragile X repeat would be consistent with the idea that the repeats themselves are the site of nucleation for the silencing process (Kumari and Usdin, 2010). *FMR1* mRNA knockdown blocks *FMR1* gene silencing during neuronal differentiation in fragile X embryonic stem cells (Colak et al., 2014) and decreases the recruitment of polycomb repressive complex 2 (PRC2) to full mutation alleles that have been reactivated with 5-azadeoxycytidine (Kumari and Usdin, 2014). These findings suggest that the *FMR1* transcript plays a key role in gene silencing by facilitating, either directly or indirectly, the recruitment of repressive histone modifying complexes like PRC2.

The *FMR1* transcript may facilitate gene silencing by recruiting repressive histone modifiers as many long non-coding RNAs do. Examples are known where such RNA acts in *cis* or *trans* to recruit PRC2 along with either the LSD1-CoREST complexes responsible for H3K4me2 demethylation (Tsai et al., 2010), or the polycomb repressive complex 1 (Yap et al., 2010), or the H3K9 methylase G9a (Pandey et al., 2008). Long non-coding RNAs are also involved in recruiting the H4K20 trimethylase Suv4-20h (Bierhoff et al., 2014). The *FMR1* transcript may be acting like these RNAs to recruit repressors to the fragile X locus. These factors may in turn act as a molecular scaffold to which other chromatin modifiers bind to ultimately generate the histone modification signature found on fragile X alleles (Kumari and Usdin, 2010). Of interest in this regard is the fact that the *Fmr1* transcript is one of the transcripts most commonly associated with PRC2 in normal mouse embryonic stem cells (Zhao et al., 2010). This may be related to the ability of the 5′ end of the transcript to form stem-loop structures as assessed by the RNAfold algorithm (http://rna.tbi.univie.ac.at/cgi-bin/RNAfold.cgi). Such structures are thought to be important for PRC2 binding (Zhao et al., 2008). The human *FMR1* transcript is predicted to form similar structures and may thus also recruit PRC2 to even to normal and premutation alleles. However, not only would the amount of PRC2 recruited to the *FMR1* locus be limited by the relatively low stability of the R loop on small repeat tracts, but any activity of PRC2 would be also inhibited by the presence of nascent RNA (Cifuentes-Rojas et al., 2014) or H3K36me3 (Schmitges et al., 2011), a histone modification deposited co-transcriptionally by the SETD2 protein. Even should some trimethylation of H3K27 occur, it’s spread would be limited by the high levels of H3K36me3 present in the region (Yuan et al., 2011).

In some cases targeting of the long non-coding RNA is thought to be accomplished by a bivalent protein that binds both the RNA and the target locus (Jeon and Lee, 2011), whilst in others the RNA is tethered to its target gene via the formation of a RNA:DNA:DNA triplex (Schmitz et al., 2010), or an RNA: DNA hybrid (Rinn and Chang, 2012). Tethering via the formation of an RNA:RNA hybrid has also been suggested (Rinn and Chang, 2012). Recruitment of repressive complexes via an RNA:DNA hybrid may be relevant for *FMR1* gene silencing given the persistent R loop that is found coincident with the repeat on *FMR1* alleles (Groh et al., 2014; Loomis et al., 2014) and the effect of R loop depletion on gene silencing in fragile X ESC-derived neurons (Colak et al., 2014). R loops have also been implicated in gene silencing in Friedreich ataxia (Groh et al., 2014). In principle, either a *cis* or *trans*-association of the transcript with the *FMR1* locus could tether the transcript to the promoter allowing it to recruit repressive epigenetic modifiers like PRC2 that ultimately result in *FMR1* gene silencing.

Gene silencing may also be triggered by the R loops themselves in ways that are independent of the *FMR1* transcript per se. The free DNA strand in the fragile X R loop forms secondary structures (Loomis et al., 2014) that have been suggested to directly recruit DNA methyltransferases (Smith et al., 1994). However, our data suggests that DNA methylation is not the first step in the fragile X gene silencing process (Biacsi et al., 2008; Kumari and Usdin, 2014). Furthermore, Friedreich ataxia repeats do not form such secondary structures and methylatable CpG residues are not present in either the Friedreich ataxia repeats or the myotonic dystrophy type 1 repeats. Thus if gene silencing in these disorders has a common molecular basis, the use of this pathway to initiate gene silencing seems unlikely. R loops also facilitate Pol II pausing and termination (Skourti-Stathaki et al., 2011; Groh et al., 2014). In the β-actin locus pausing results in the production of an antisense transcript. The sense-antisense RNA pair leads to the RNAi-dependent deposition of H3K9me2 and the recruitment of heterochromatin protein 1*γ* (HP1*γ*; Skourti-Stathaki et al., 2014). However, knockdown of *Dicer*, *Ago1*, and *Ago2*, genes important for *RNAi* did not prevent *FMR1* gene silencing in neurons differentiated from fragile X embryonic stem cells (Colak et al., 2014). This would seem to rule out a role for RNAi in silencing of full mutation alleles, whether the RNAi is R loop-mediated or mediated independently of R loops by the interaction of the FMR1 sense and antisense transcript (Ladd et al., 2007) or CGG-RNA hairpins that are Dicer substrates (Handa et al., 2003). R loops are also prone to chromatin compaction via a H3S10 phosphorylation-dependent mechanism that is still unknown (Castellano-Pozo et al., 2013). R loops have also been shown to result in double-strand breaks (Sordet et al., 2010) that could lead to the recruitment of a variety of epigenetic modifiers including DNA methyltransferase 1 and SIRT1 (O’Hagan et al., 2008).

## Unresolved Questions and Future Directions

**FIGURE 1:**
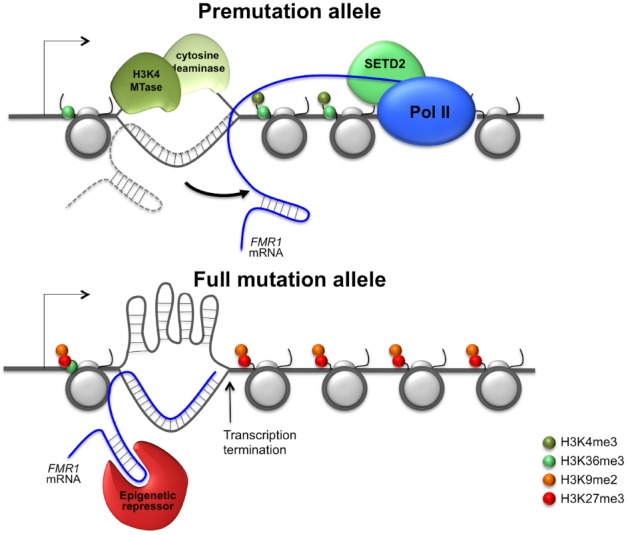
**Model for repeat-mediated gene dysregulation in fragile X premutation and full mutation carriers.** A metastable R loop formed by the premutation allele would leave the non-template strand transiently unpaired and thus able to recruit transcription activators that show a preferential binding to single-stranded regions (Chaudhuri et al., 2003; Krajewski et al., 2005). However, since the RNA:DNA hybrid is relatively short, transcription termination is low and the transcript is not tethered to the *FMR1* locus long enough to recruit transcriptional repressors that might bind the hairpins formed by the 5′ end of the *FMR1* transcript. The net result is that the premutation allele would be associated with elevated levels of active histone modifications and thus hyper-expressed. In contrast, on full mutation alleles the RNA:DNA hybrid is likely to be more stable . It may thus be able to effectively recruit repressive chromatin modifiers to the 5′ end of the *FMR1* gene that result in the deposition of repressive histone marks. The hybrid may also be long enough to cause significant transcription termination (Skourti-Stathaki et al., 2011; Groh et al., 2014). This would result in a drop in the levels of co-transcriptionally deposited active chromatin modifications. This could result in the loss of the protective effect that these histone marks provide against the deposition of repressive histone marks (Schmitges et al., 2011; Yuan et al., 2011). The non-template strand in the R loop may also be more likely to form secondary structures and thus less likely to bind transcription activators with a preference for single-stranded regions. The net result would be transcriptional silencing of the full mutation allele.

More work is needed before we understand the epigenetic regulation of the *FMR1* gene. Until we do the question of why shorter repeat tracts favor increased gene expression while longer ones favor gene silencing remains unanswered. The paradoxical effect of the repeats presumably reflects the equilibrium between interactions of the repeat tract with factors that favor the accumulation of positive chromatin modifications and those that favor the generation of repressive chromatin. For example, as illustrated in Figure 1, smaller repeat tracts may be more likely to form RNA: DNA hybrids that have a short half-life and that may leave the non-template strand single-stranded just long enough to bind transcriptional activators or DNA demethylases that have a preference for single-stranded regions. In contrast, longer repeat tracts would be more likely to form stable RNA:DNA hybrids that were better able to recruit complexes like G9a and PRC2 that deposit repressive chromatin marks. The longer hybrids may also be more prone to transcription termination that could result in the loss of H3K36me3 and H3K4me3 and thus the protection from encroachment of repressive chromatin (Yuan et al., 2011). The non-template strand may also be more likely to form stable secondary structures that reduce binding of transcription activators with a preference for single-stranded regions.

In addition, while the idea that a co-transcriptionally formed R loop is responsible for silencing is appealing, the ability of siRNA or shRNA to the *FMR1* transcript to reduce repression of fragile X alleles (Colak et al., 2014; Kumari and Usdin, 2014) suggests that the situation may not be quite so simple. Despite reports of active RNAi factors in the nucleus (Gagnon et al., 2014), the ability of RNAi to deplete RNAs that are exclusively nuclear is still controversial. An alternative explanation consistent with the available data is that the R loop forms *in trans* from a transcript that has transitioned through the cytoplasm. If so, then R loop formation may be facilitated by the ability of the CGG-strand of the DNA to form secondary structures during transcription thus leaving the CCG-DNA strand free.

The question of the timing of gene silencing in the fragile X embryo is also still unresolved. This is significant for our understanding as to whether FMRP is present during early embryonic development in individuals with fragile X syndrome. Silencing has been reported to be a relatively late event in embryonic development, occurring >45 days after differentiation of neurons from embryonic stem cells is initiated (Colak et al., 2014). However, in many fragile X ESC lines significant gene silencing has already occurred (Avitzour et al., 2014). This includes the ESC lines used in Colak et. al. study as evidenced by the fact that a significant fraction of the fragile X alleles in these cells are resistant to digestion by *Eag I*, a methylation-sensitive restriction enzyme (Supplementary Figure 1; Colak et al., 2014). How much silencing occurs in the early embryo and how this silencing compares to the silencing observed in neurons are thus important open questions.

There is also the question of what, if anything, can be done to prevent or reverse the repeat-mediated epigenetic changes in premutation and full mutation carriers. A number of compounds that are able to inhibit repressive epigenetic modifications are already in clinical trials for treatment of Friedreich ataxia. It is of interest that Vitamin B3 (nicotinamide), an inhibitor of SIRT1, has shown promise in Phase I trials for Friedreich ataxia (Libri et al., 2014). The identification of *FMR1* transcription as the trigger for silencing opens up a whole new potential approach to gene reactivation. A small molecule that blocks hybrid formation and prevents silencing (Colak et al., 2014) was unable to reactivate a silenced allele. However, it may able to do so in combination with other epigenetic modifiers. Of course, any benefit to be gained by reactivation of the fragile X allele would have to be offset by the risk posed by expression of *FMR1* mRNA with long CGG-repeat tracts (Loesch et al., 2012). It may also be possible to ameliorate the symptoms seen in premutation carriers by using epigenetic modifying compounds that reduce the *FMR1* hyper-expression. For example, since SIRT1 is involved in down regulation of *FMR1* expression, SIRT1 activators may help normalize the levels of the toxic transcript produced from premutation alleles.

### Conflict of Interest Statement

The authors declare that the research was conducted in the absence of any commercial or financial relationships that could be construed as a potential conflict of interest.

## References

[B1] Avitzour M. Mor-Shaked H. Yanovsky-Dagan S. Aharoni S. Altarescu G. Renbaum P. (2014). *FMR1* epigenetic silencing commonly occurs in undifferentiated fragile X-affected embryonic stem cells. Stem Cell Reports 3, 699–706. 10.1016/j.stemcr.2014.09.001 25418717 PMC4235235

[B2] Beilina A. Tassone F. Schwartz P. H. Sahota P. Hagerman P. J. (2004). Redistribution of transcription start sites within the *FMR1* promoter region with expansion of the downstream CGG-repeat element. Hum. Mol. Genet. 13, 543–549. 10.1093/hmg/ddh053 14722156

[B3] Belzil V. V. Bauer P. O. Prudencio M. Gendron T. F. Stetler C. T. Yan I. K. (2013). Reduced *C9orf72* gene expression in c9FTD/ALS is caused by histone trimethylation, an epigenetic event detectable in blood. Acta Neupathol. 126, 895–905. 10.1007/s00401-013-1199-1 24166615 PMC3830740

[B4] Biacsi R. Kumari D. Usdin K. (2008). SIRT1 inhibition alleviates gene silencing in fragile X mental retardation syndrome. PLoS Genet. 4:e1000017. 10.1371/journal.pgen.1000017 18369442 PMC2265469

[B5] Bierhoff H. Dammert M. A. Brocks D. Dambacher S. Schotta G. Grummt I. (2014). Quiescence-induced LncRNAs trigger H4K20 trimethylation and transcriptional silencing. Mol. Cell 54, 675–682. 10.1016/j.molcel.2014.03.032 24768537

[B6] Blackledge N. P. Thomson J. P. Skene P. J. (2013). CpG island chromatin is shaped by recruitment of ZF-CxxC proteins. Cold Spring Harb. Perspect. Biol. 5, a018648. 10.1101/cshperspect.a018648 24186071 PMC3809580

[B7] Brouwer J. R. Huizer K. Severijnen L. A. Hukema R. K. Berman R. F. Oostra B. A. (2008). CGG-repeat length and neuropathological and molecular correlates in a mouse model for fragile X-associated tremor/ataxia syndrome. J. Neurochem. 107, 1671–1682. 10.1111/j.1471-4159.2008.05747.x 19014369 PMC2605773

[B8] Castellano-Pozo M. Santos-Pereira J. M. Rondon A. G. Barroso S. Andujar E. Perez-Alegre M. (2013). R loops are linked to histone H3 S10 phosphorylation and chromatin condensation. Mol. Cell 52, 583–590. 10.1016/j.molcel.2013.10.006 24211264

[B9] Chaudhuri J. Tian M. Khuong C. Chua K. Pinaud E. Alt F. W. (2003). Transcription-targeted DNA deamination by the AID antibody diversification enzyme. Nature 422, 726–730. 10.1038/nature01574 12692563

[B10] Chiurazzi P. Pomponi M. G. Pietrobono R. Bakker C. E. Neri G. Oostra B. A. (1999). Synergistic effect of histone hyperacetylation and DNA demethylation in the reactivation of the *FMR1* gene. Hum. Mol. Genet. 8, 2317–2323. 10.1093/hmg/8.12.2317 10545613

[B11] Chiurazzi P. Pomponi M. G. Willemsen R. Oostra B. A. Neri G. (1998). *In vitro* reactivation of the *FMR1* gene involved in fragile X syndrome. Hum. Mol. Genet. 7, 109–113. 10.1093/hmg/7.1.109 9384610

[B12] Cho D. H. Thienes C. P. Mahoney S. E. Analau E. Filippova G. N. Tapscott S. J. (2005). Antisense transcription and heterochromatin at the *DM1* CTG repeats are constrained by CTCF. Mol. Cell 20, 483–489. 10.1016/j.molcel.2005.09.002 16285929

[B13] Cifuentes-Rojas C. Hernandez A. J. Sarma K. Lee J. T. (2014). Regulatory interactions between RNA and polycomb repressive complex 2. Mol. Cell 55, 171–185. 10.1016/j.molcel.2014.05.009 24882207 PMC4107928

[B14] Clouaire T. Webb S. Skene P. Illingworth R. Kerr A. Andrews R. (2012). Cfp1 integrates both CpG content and gene activity for accurate H3K4me3 deposition in embryonic stem cells. Genes Dev. 26, 1714–1728. 10.1101/gad.194209.112 22855832 PMC3418589

[B15] Coffee B. Zhang F. Warren S. T. Reines D. (1999). Acetylated histones are associated with *FMR1* in normal but not fragile X-syndrome cells. Nat. Genet. 22, 98–101. 10319871 10.1038/8807

[B16] Colak D. Zaninovic N. Cohen M. S. Rosenwaks Z. Yang W. Y. Gerhardt J. (2014). Promoter-bound trinucleotide repeat mRNA drives epigenetic silencing in fragile X syndrome. Science 343, 1002–1005. 10.1126/science.1245831 24578575 PMC4357282

[B17] Dunn K. Griffith J. D. (1980). The presence of RNA in a double helix inhibits its interaction with histone proteins. Nucl. Acids Res. 11, 555–566. 6160477 10.1093/nar/8.3.555 PMC327289

[B18] Entezam A. Biacsi R. Orrison B. Saha T. Hoffman G. E. Grabczyk E. (2007). Regional FMRP deficits and large repeat expansions into the full mutation range in a new fragile X premutation mouse model. Gene 395, 125–134. 10.1016/j.gene.2007.02.026 17442505 PMC1950257

[B19] Fratta P. Mizielinska S. Nicoll A. J. Zloh M. Fisher E. M. Parkinson G. (2012). C9orf72 hexanucleotide repeat associated with amyotrophic lateral sclerosis and frontotemporal dementia forms RNA G-quadruplexes. Sci. Rep. 2, 1016. 10.1038/srep01016 23264878 PMC3527825

[B20] Fry M. Loeb L. A. (1994). The fragile X syndrome d(CGG)n nucleotide repeats form a stable tetrahelical structure. Proc. Natl. Acad. Sci. U.S.A. 91, 4950–4954. 8197163 10.1073/pnas.91.11.4950 PMC43907

[B21] Fu Y. H. Kuhl D. P. Pizzuti A. Pieretti M. Sutcliffe J. S. Richards S. (1991). Variation of the CGG repeat at the fragile X site results in genetic instability: resolution of the Sherman paradox. Cell 67, 1047–1058. 1760838 10.1016/0092-8674(91)90283-5

[B22] Gagnon K. T. Li L. Chu Y. Janowski B. A. Corey D. R. (2014). RNAi factors are present and active in human cell nuclei. Cell Rep. 6, 211–221. 10.1016/j.celrep.2013.12.013 24388755 PMC3916906

[B23] Ginno P. A. Lott P. L. Christensen H. C. Korf I. Chedin F. (2012). R-loop formation is a distinctive characteristic of unmethylated human CpG island promoters. Mol. Cell 45, 814–825. 10.1016/j.molcel.2012.01.017 22387027 PMC3319272

[B24] Greene E. Mahishi L. Entezam A. Kumari D. Usdin K. (2007). Repeat-induced epigenetic changes in intron 1 of the frataxin gene and its consequences in Friedreich ataxia. Nucleic Acids Res. 35, 3383–3390. 10.1093/nar/gkm271 17478498 PMC1904289

[B25] Groh M. Lufino M. M. Wade-Martins R. Gromak N. (2014). R-loops associated with triplet repeat expansions promote gene silencing in Friedreich ataxia and fragile X syndrome. PLoS Genet. 10:e1004318. 10.1371/journal.pgen.1004318 24787137 PMC4006715

[B26] Haeusler A. R. Donnelly C. J. Periz G. Simko E. A. Shaw P. G. Kim M. S. (2014). C9orf72 nucleotide repeat structures initiate molecular cascades of disease. Nature 507, 195–200. 10.1038/nature13124 24598541 PMC4046618

[B27] Hagerman P. (2013). Fragile X-associated tremor/ataxia syndrome (FXTAS): pathology and mechanisms. Acta Neuropathol. 126, 1–19. 10.1007/s00401-013-1138-1 23793382 PMC3904666

[B28] Handa V. Saha T. Usdin K. (2003). The fragile X syndrome repeats form RNA hairpins that do not activate the interferon-inducible protein kinase, PKR, but are cut by Dicer. Nucleic Acids Res. 31, 6243–6248. 10.1093/nar/gkg818 14576312 PMC275460

[B29] Herman D. Jenssen K. Burnett R. Soragni E. Perlman S. L. Gottesfeld J. M. (2006). Histone deacetylase inhibitors reverse gene silencing in Friedreich’s ataxia. Nat. Chem. Biol. 2, 551–558. 10.1038/nchembio815 16921367

[B30] Jeon Y. Lee J. T. (2011). YY1 tethers Xist RNA to the inactive X nucleation center. Cell 146, 119–133. 10.1016/j.cell.2011.06.026 21729784 PMC3150513

[B31] Kettani A. Kumar R. A. Patel D. J. (1995). Solution structure of a DNA quadruplex containing the fragile X syndrome triplet repeat. J. Mol. Biol. 254, 638–656. 7500339 10.1006/jmbi.1995.0644

[B32] Krajewski W. A. Nakamura T. Mazo A. Canaani E. (2005). A motif within SET-domain proteins binds single-stranded nucleic acids and transcribed and supercoiled DNAs and can interfere with assembly of nucleosomes. Mol. Cell. Biol. 25, 1891–1899. 10.1128/MCB.25.5.1891-1899.2005 15713643 PMC549386

[B33] Kumari D. Usdin K. (2010). The distribution of repressive histone modifications on silenced *FMR1* alleles provides clues to the mechanism of gene silencing in fragile X syndrome. Hum. Mol. Genet. 19, 4634–4642. 10.1093/hmg/ddq394 20843831 PMC2972696

[B34] Kumari D. Usdin K. (2014). Polycomb group complexes are recruited to reactivated *FMR1* alleles in Fragile X syndrome in response to *FMR1* transcription. Hum. Mol. Genet. 23, 6575–6583. 10.1093/hmg/ddu378 25055869 PMC4240206

[B35] Ladd P. D. Smith L. E. Rabaia N. A. Moore J. M. Georges S. A. Hansen R. S. (2007). An antisense transcript spanning the CGG repeat region of *FMR1* is upregulated in premutation carriers but silenced in full mutation individuals. Hum. Mol. Genet. 16, 3174–3187. 10.1093/hmg/ddm293 17921506

[B36] Levy M. A. Kernohan K. D. Jiang Y. Berube N. G. (2015). ATRX promotes gene expression by facilitating transcriptional elongation through guanine-rich coding regions. Hum. Mol. Genet. 24, 1824–1835. 10.1093/hmg/ddu596 25452430

[B37] Libri V. Yandim C. Athanasopoulos S. Loyse N. Natisvili T. Law P. P. (2014). Epigenetic and neurological effects and safety of high-dose nicotinamide in patients with Friedreich’s ataxia: an exploratory, open-label, dose-escalation study. Lancet 384, 504–513. 10.1016/S0140-6736(14)60382-2 24794816

[B38] Lin Y. Dent S. Y. Wilson J. H. Wells R. D. Napierala M. (2010). R loops stimulate genetic instability of CTG.CAG repeats. Proc. Natl. Acad. Sci. U.S.A. 107, 692–697. 10.1073/pnas.0909740107 20080737 PMC2818888

[B39] Liu G. Chen X. Bissler J. J. Sinden R. R. Leffak M. (2010). Replication-dependent instability at (CTG) x (CAG) repeat hairpins in human cells. Nat. Chem. Biol. 6, 652–659. 10.1038/nchembio.416 20676085 PMC2924473

[B40] Loesch D. Z. Sherwell S. Kinsella G. Tassone F. Taylor A. Amor D. (2012). Fragile X-associated tremor/ataxia phenotype in a male carrier of unmethylated full mutation in the *FMR1* gene. Clin. Genet. 82, 88–92. 10.1111/j.1399-0004.2011.01675.x 21476992

[B41] Loomis E. W. Sanz L. A. Chedin F. Hagerman P. J. (2014). Transcription-associated R-loop formation across the human *FMR1* CGG-repeat region. PLoS Genet. 10:e1004294. 10.1371/journal.pgen.1004294 24743386 PMC3990486

[B42] Malgowska M. Gudanis D. Kierzek R. Wyszko E. Gabelica V. Gdaniec Z. (2014). Distinctive structural motifs of RNA G-quadruplexes composed of AGG, CGG and UGG trinucleotide repeats. Nucleic Acids Res. 42, 10196–10207. 10.1093/nar/gku710 25081212 PMC4150804

[B43] Mirkin S. M. (2007). Expandable DNA repeats and human disease. Nature 447, 932–940. 10.1038/nature05977 17581576

[B44] Mitas M. Yu A. Dill J. Haworth I. S. (1995). The trinucleotide repeat sequence d(CGG)15 forms a heat-stable hairpin containing Gsyn. Ganti base pairs. Biochemistry 34, 12803–12811. 7548035 10.1021/bi00039a041

[B45] O’Hagan H. M. Mohammad H. P. Baylin S. B. (2008). Double strand breaks can initiate gene silencing and SIRT1-dependent onset of DNA methylation in an exogenous promoter CpG island. PLoS Genet. 4:e1000155. 10.1371/journal.pgen.1000155 18704159 PMC2491723

[B46] Oberle I. Rousseau F. Heitz D. Kretz C. Devys D. Hanauer A. (1991). Instability of a 550-base pair DNA segment and abnormal methylation in fragile X syndrome. Science 252, 1097–1102. 10.1126/science.252.5009.1097 2031184

[B47] Ooi S. K. Qiu C. Bernstein E. Li K. Jia D. Yang Z. (2007). DNMT3L connects unmethylated lysine 4 of histone H3 to *de novo* methylation of DNA. Nature 448, 714–717. 10.1038/nature05987 17687327 PMC2650820

[B48] Otten A. D. Tapscott S. J. (1995). Triplet repeat expansion in myotonic dystrophy alters the adjacent chromatin structure. Proc. Natl. Acad. Sci. U.S.A. 92, 5465–5469. 7777532 10.1073/pnas.92.12.5465 PMC41715

[B49] Pandey R. R. Mondal T. Mohammad F. Enroth S. Redrup L. Komorowski J. (2008). Kcnq1ot1 antisense noncoding RNA mediates lineage-specific transcriptional silencing through chromatin-level regulation. Mol. Cell 32, 232–246. 10.1016/j.molcel.2008.08.022 18951091

[B50] Patel P. K. Bhavesh N. S. Hosur R. V. (2000). Cation-dependent conformational switches in d-TGGCGGC containing two triplet repeats of Fragile X Syndrome: NMR observations. Biochem. Biophys. Res. Commun. 278, 833–838. 10.1006/bbrc.2000.3878 11095993

[B51] Pieretti M. Zhang F. P. Fu Y. H. Warren S. T. Oostra B. A. Caskey C. T. (1991). Absence of expression of the *FMR-1* gene in fragile X syndrome. Cell 66, 817–822. 1878973 10.1016/0092-8674(91)90125-i

[B52] Pietrobono R. Pomponi M. G. Tabolacci E. Oostra B. Chiurazzi P. Neri G. (2002). Quantitative analysis of DNA demethylation and transcriptional reactivation of the *FMR1* gene in fragile X cells treated with 5-azadeoxycytidine. Nucleic Acids Res. 30, 3278–3285. 10.1093/nar/gkf434 12136110 PMC135754

[B53] Pietrobono R. Tabolacci E. Zalfa F. Zito I. Terracciano A. Moscato U. (2005). Molecular dissection of the events leading to inactivation of the *FMR1* gene. Hum. Mol. Genet. 14, 267–277. 10.1093/hmg/ddi024 15563507

[B54] Popp C. Dean W. Feng S. Cokus S. J. Andrews S. Pellegrini M. (2010). Genome-wide erasure of DNA methylation in mouse primordial germ cells is affected by AID deficiency. Nature 463, 1101–1105. 10.1038/nature08829 20098412 PMC2965733

[B55] Powell W. T. Coulson R. L. Gonzales M. L. Crary F. K. Wong S. S. Adams S. (2013). R-loop formation at Snord116 mediates topotecan inhibition of Ube3a-antisense and allele-specific chromatin decondensation. Proc. Natl. Acad. Sci. U.S.A. 110, 13938–13943. 10.1073/pnas.1305426110 23918391 PMC3752217

[B56] Reddy K. Zamiri B. Stanley S. Y. Macgregor R. B. Jr. Pearson C. E. (2013). The disease-associated r(GGGGCC)n repeat from the C9orf72 gene forms tract length-dependent uni- and multimolecular RNA G-quadruplex structures. J. Biol. Chem. 288, 9860–9866. 10.1074/jbc.C113.452532 23423380 PMC3617286

[B57] Rinn J. L. Chang H. Y. (2012). Genome regulation by long noncoding RNAs. Annu. Rev. Biochem. 81, 145–166. 10.1146/annurev-biochem-051410-092902 22663078 PMC3858397

[B58] Saha T. Usdin K. (2001). Tetraplex formation by the progressive myoclonus epilepsy type-1 repeat: implications for instability in the repeat expansion diseases. FEBS Lett. 491, 184–187. 10.1016/S0014-5793(01)02190-1 11240124

[B59] Sareen D. O’rourke J. G. Meera P. Muhammad A. K. Grant S. Simpkinson M. (2013). Targeting RNA foci in iPSC-derived motor neurons from ALS patients with a *C9ORF72* repeat expansion. Sci. Transl. Med. 5, 208ra149. 10.1126/scitranslmed.3007529 24154603 PMC4090945

[B60] Schmitges F. W. Prusty A. B. Faty M. Stutzer A. Lingaraju G. M. Aiwazian J. (2011). Histone methylation by PRC2 is inhibited by active chromatin marks. Mol. Cell. 42, 330–341. 10.1016/j.molcel.2011.03.025 21549310

[B61] Schmitz K. M. Mayer C. Postepska A. Grummt I. (2010). Interaction of noncoding RNA with the rDNA promoter mediates recruitment of DNMT3b and silencing of rRNA genes. Genes Dev. 24, 2264–2269. 10.1101/gad.590910 20952535 PMC2956204

[B62] Sellier C. Usdin K. Pastori C. Peschansky V. J. Tassone F. Charlet-Berguerand N. (2014). The multiple molecular facets of fragile X-associated tremor/ataxia syndrome. J. Neurodev. Disord. 6, 23. 10.1186/1866-1955-6-23 25161746 PMC4144988

[B63] Sherman S. L. Curnow E. C. Easley C. A. Jin P. Hukema R. K. Tejada M. I. (2014). Use of model systems to understand the etiology of fragile X-associated primary ovarian insufficiency (FXPOI). J. Neurodev. Disord. 6, 26. 10.1186/1866-1955-6-26 25147583 PMC4139715

[B64] Skourti-Stathaki K. Kamieniarz-Gdula K. Proudfoot N. J. (2014). R-loops induce repressive chromatin marks over mammalian gene terminators. Nature 516, 436–439. 10.1038/nature13787 25296254 PMC4272244

[B65] Skourti-Stathaki K. Proudfoot N. J. Gromak N. (2011). Human senataxin resolves RNA/DNA hybrids formed at transcriptional pause sites to promote Xrn2-dependent termination. Mol. Cell 42, 794–805. 10.1016/j.molcel.2011.04.026 21700224 PMC3145960

[B66] Smith S. S. Laayoun A. Lingeman R. G. Baker D. J. Riley J. (1994). Hypermethylation of telomere-like foldbacks at codon 12 of the human c-Ha-ras gene and the trinucleotide repeat of the FMR-1 gene of fragile X. J. Mol. Biol. 243, 143–151. 10.1006/jmbi.1994.1640 7932745

[B67] Sordet O. Nakamura A. J. Redon C. E. Pommier Y. (2010). DNA double-strand breaks and ATM activation by transcription-blocking DNA lesions. Cell Cycle 9, 274–278. 10.4161/cc.9.2.10506 20023421 PMC7289056

[B68] Steinbach P. Glaser D. Vogel W. Wolf M. Schwemmle S. (1998). The DMPK gene of severely affected myotonic dystrophy patients is hypermethylated proximal to the largely expanded CTG repeat. Am. J. Hum. Genet. 62, 278–285. 10.1086/301711 9463318 PMC1376887

[B69] Sullivan S. D. Welt C. Sherman S. (2011). *FMR1* and the continuum of primary ovarian insufficiency. Semin. Reprod. Med. 29, 299–307. 10.1055/s-0031-1280915 21969264

[B70] Sutcliffe J. S. Nelson D. L. Zhang F. Pieretti M. Caskey C. T. Saxe D. (1992). DNA methylation represses FMR-1 transcription in fragile X syndrome. Hum. Mol. Genet. 1, 397–400. 1301913 10.1093/hmg/1.6.397

[B71] Tabolacci E. Moscato U. Zalfa F. Bagni C. Chiurazzi P. Neri G. (2008). Epigenetic analysis reveals a euchromatic configuration in the *FMR1* unmethylated full mutations. Eur. J. Hum. Genet. 16, 1487–1498. 10.1038/ejhg.2008.130 18628788

[B72] Tassone F. Beilina A. Carosi C. Albertosi S. Bagni C. Li L. (2007). Elevated *FMR1* mRNA in premutation carriers is due to increased transcription. RNA 13, 555–562. 10.1261/rna.280807 17283214 PMC1831862

[B73] Tassone F. Hagerman R. J. Loesch D. Z. Lachiewicz A. Taylor A. K. Hagerman P. J. (2000a). Fragile X males with unmethylated, full mutation trinucleotide repeat expansions have elevated levels of *FMR1* messenger RNA. Am. J. Med. Genet. 94, 232–236. 10.1002/1096-8628(20000918)94:3<232::AID-AJMG9>3.0.CO;2-H 10995510

[B74] Tassone F. Hagerman R. J. Taylor A. K. Gane L. W. Godfrey T. E. Hagerman P. J. (2000b). Elevated levels of *FMR1* mRNA in carrier males: a new mechanism of involvement in the fragile-X syndrome. Am. J. Hum. Genet. 66, 6–15. 10.1086/302720 10631132 PMC1288349

[B75] Thomson J. P. Skene P. J. Selfridge J. Clouaire T. Guy J. Webb S. (2010). CpG islands influence chromatin structure via the CpG-binding protein Cfp1. Nature 464, 1082–1086. 10.1038/nature08924 20393567 PMC3730110

[B76] Todd P. K. Oh S. Y. Krans A. Pandey U. B. Di Prospero N. A. Min K. T. (2010). Histone deacetylases suppress CGG repeat-induced neurodegeneration via transcriptional silencing in models of fragile X tremor ataxia syndrome. PLoS Genet. 6:e1001240. 10.1371/journal.pgen.1001240 21170301 PMC3000359

[B77] Tsai M. C. Manor O. Wan Y. Mosammaparast N. Wang J. K. Lan F. (2010). Long noncoding RNA as modular scaffold of histone modification complexes. Science 329, 689–693. 10.1126/science.1192002 20616235 PMC2967777

[B78] Usdin K. House N. C. Freudenreich C. H. (2015). Repeat instability during DNA repair: insights from model systems. Crit. Rev. Biochem. Mol. Biol. 50, 142–167. 10.3109/10409238.2014.999192 25608779 PMC4454471

[B79] Usdin K. Woodford K. J. (1995). CGG repeats associated with DNA instability and chromosome fragility form structures that block DNA synthesis *in vitro*. Nucleic Acids Res. 23, 4202–4209. 7479085 10.1093/nar/23.20.4202 PMC307363

[B80] Verkerk A. J. Pieretti M. Sutcliffe J. S. Fu Y. H. Kuhl D. P. Pizzuti A. (1991). Identification of a gene (FMR-1) containing a CGG repeat coincident with a breakpoint cluster region exhibiting length variation in fragile X syndrome. Cell 65, 905–914. 1710175 10.1016/0092-8674(91)90397-h

[B81] Wahba L. Koshland D. (2013). The Rs of biology: R-loops and the regulation of regulators. Mol. Cell 50, 611–612. 10.1016/j.molcel.2013.05.024 23746348

[B82] Wang Y. H. Gellibolian R. Shimizu M. Wells R. D. Griffith J. (1996). Long CCG triplet repeat blocks exclude nucleosomes: a possible mechanism for the nature of fragile sites in chromosomes. J. Mol. Biol. 263, 511–516. 10.1006/jmbi.1996.0593 8918933

[B83] Weiler I. J. Greenough W. T. (1999). Synaptic synthesis of the Fragile X protein: possible involvement in synapse maturation and elimination. Am. J. Med. Genet. 83, 248–252. 10208156 10.1002/(sici)1096-8628(19990402)83:4<248::aid-ajmg3>3.0.co;2-1

[B84] Weiler I. J. Irwin S. A. Klintsova A. Y. Spencer C. M. Brazelton A. D. Miyashiro K. (1997). Fragile X mental retardation protein is translated near synapses in response to neurotransmitter activation. Proc. Natl. Acad. Sci. U.S.A. 94, 5395–5400. 9144248 10.1073/pnas.94.10.5395 PMC24689

[B85] Xi Z. Zinman L. Moreno D. Schymick J. Liang Y. Sato C. (2013). Hypermethylation of the CpG island near the G4C2 repeat in ALS with a C9orf72 expansion. Am. J. Hum. Genet. 92, 981–989. 10.1016/j.ajhg.2013.04.017 23731538 PMC3675239

[B86] Yap K. L. Li S. Munoz-Cabello A. M. Raguz S. Zeng L. Mujtaba S. (2010). Molecular interplay of the noncoding RNA ANRIL and methylated histone H3 lysine 27 by polycomb CBX7 in transcriptional silencing of INK4a. Mol. Cell 38, 662–674. 10.1016/j.molcel.2010.03.021 20541999 PMC2886305

[B87] Yuan W. Xu M. Huang C. Liu N. Chen S. Zhu B. (2011). H3K36 methylation antagonizes PRC2 mediated H3K27 methylation. J. Biol. Chem. 286, 7983–7989. 10.1074/jbc.M110.194027 21239496 PMC3048685

[B88] Zaitsev E. N. Kowalczykowski S. C. (2000). A novel pairing process promoted by *Escherichia coli* RecA protein: inverse DNA and RNA strand exchange. Genes Dev. 14, 740–749. 10733533 PMC316457

[B89] Zhao J. Ohsumi T. K. Kung J. T. Ogawa Y. Grau D. J. Sarma K. (2010). Genome-wide identification of polycomb-associated RNAs by RIP-seq. Mol. Cell 40, 939–953. 10.1016/j.molcel.2010.12.011 21172659 PMC3021903

[B90] Zhao J. Sun B. K. Erwin J. A. Song J. J. Lee J. T. (2008). Polycomb proteins targeted by a short repeat RNA to the mouse X chromosome. Science 322, 750–756. 10.1126/science.1163045 18974356 PMC2748911

[B91] Zumwalt M. Ludwig A. Hagerman P. J. Dieckmann T. (2007). Secondary structure and dynamics of the r(CGG) repeat in the mRNA of the fragile X mental retardation 1 (*FMR1*) gene. RNA Biol. 4, 93–100. 10.4161/rna.4.2.5039 17962727

